# Research on a Hybrid Self-Powered Landslide Rainfall Sensor Based on Triboelectric Nanogenerators and Electromagnetic Generators

**DOI:** 10.3390/mi16060678

**Published:** 2025-06-04

**Authors:** Hao Zou, Zexu Zuo, Xianhong Shen, Kun Song, Shuai Mao, Bing Chen

**Affiliations:** 1 Hubei Key Laboratory of Disaster Prevention and Mitigation, China Three Gorges University, Yichang 443002, China; 2 Third Geological Brigade of Hubei Geological Bureau, Huanggang 438000, China; 18827187366@163.com (S.M.); 18671316127@163.com (B.C.); 3 Faculty of Mechanical and Electronic Information, China University of Geosciences (Wuhan), Wuhan 430074, China; 18436099151@163.com (Z.Z.); 15271506839@163.com (X.S.)

**Keywords:** triboelectric nanogenerator, self-powered, rainfall sensor, landslide

## Abstract

Landslide monitoring is crucial for mitigating landslide disaster risks. However, the power supply methods of existing rainfall sensors for landslide monitoring often fail to meet the demands of practical field applications. This study proposes a hybrid self-powered rainfall sensor for landslide monitoring, integrating a triboelectric nanogenerator (TENG) and an electromagnetic generator (EMG). The TENG module is used for rainfall monitoring, while both the TENG and EMG modules are synergistically utilized for power generation. Experimental results demonstrate that the sensor’s measurement error is less than 5%, and it can operate stably under conditions of temperature below 90 °C and humidity below 90%. Furthermore, the sensor exhibits power generation capabilities. When the TENG and EMG modules are connected to resistors of 4.3 × 10^8^ Ω and 3.6 × 10^2^ Ω, respectively, they output a maximum power of 57.5 nW and 110.25 mW, respectively. Compared to conventional rainfall sensors, this sensor is self-powered, allowing for normal operation without an external power supply, making it more suitable for field environments prone to landslides.

## 1. Introduction

Landslide monitoring is crucial for mitigating the risk of landslide disasters, but existing rainfall sensors often rely on power supply methods that are impractical for remote, field-based deployment. These disasters cause significant casualties and economic losses worldwide annually, making landslide monitoring a vital tool for risk reduction. Key parameters for landslide monitoring include rainfall [1,2], soil pressure [3], displacement [4,5], pore water pressure [6], and groundwater level [7]. Commonly used sensors and methods for monitoring these parameters include rainfall sensors [8], satellite remote sensing [9], inclinometers [10], GPS [11,12], total stations [13], and piezometers [14]. Most landslides are directly related to rainfall. Rainfall increases soil weight, reduces its shear strength, and increases pore water pressure, all of which contribute to landslide initiation. Therefore, rainfall monitoring is a crucial component of landslide prediction and early warning systems.

However, rainfall sensors for landslide monitoring are often deployed in remote and unmanned field environments. Current power supply methods for these sensors primarily rely on cable power, solar power, and battery power. However, in field environments, laying cables and replacing batteries significantly increase costs and may pose safety risks to personnel. Solar power is heavily affected by cloudy and rainy weather. Especially during critical monitoring periods when continuous rainfall is likely to trigger landslides, solar panels may experience power outages due to prolonged lack of sunlight, resulting in the loss of crucial early warning data. Thus, if a rainfall sensor could generate electricity from the rainfall environment itself, this problem could be effectively solved.

The triboelectric nanogenerator (TENG), proposed by Wang Z.L. [15] and others, has been widely utilized in the field of self-powered sensors. In the generator field, TENG can harvest energy from various sources, including wind [16,17,18], water [19], vibration [20], tides [21], and waves [22,23]. In the sensor field, TENG has been used to monitor various parameters in real time, including vibration frequency, tilt angle [24], pressure [25,26,27], velocity [28], temperature [29,30,31], and humidity [32]. Furthermore, TENG has been widely applied in downhole monitoring in the geological field. Examples include deep learning-based downhole mechanical operation monitoring [33], self-powered downhole vibration sensor research [34], and downhole energy harvesting and vibration monitoring [35].

This study proposes a hybrid TENG–electromagnetic rainfall sensor for landslide monitoring, offering dual functionality of rainfall measurement and power generation. The sensor utilizes rainfall-induced triboelectric generation to simultaneously achieve power generation and rainfall measurement. It also utilizes the impact of rainfall to drive an electromagnetic generator (EMG) to further enhance the overall power generation of the sensor.

## 2. Structure and Working Principle

**Figure 1:**
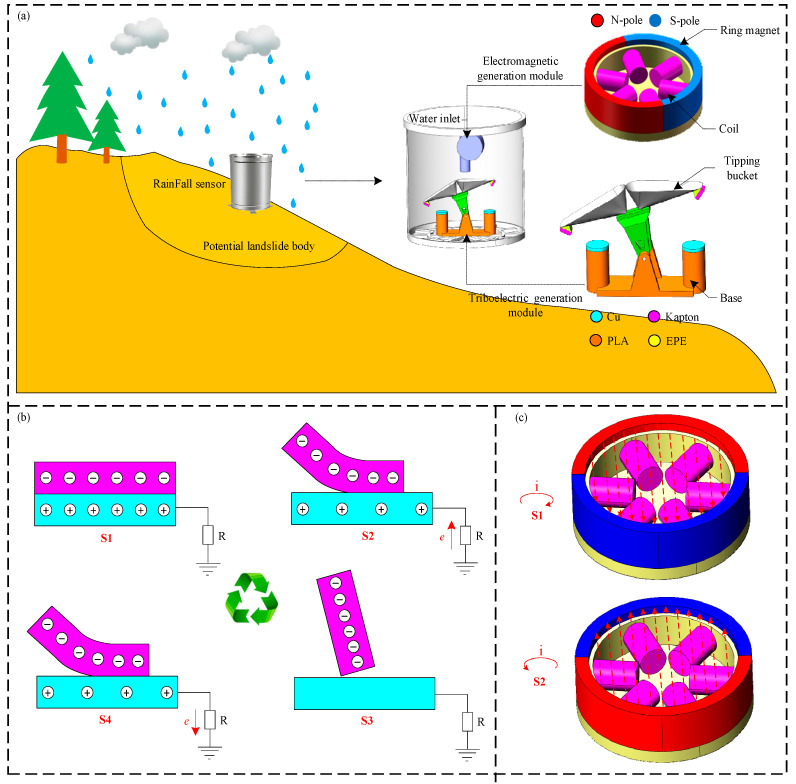
Schematic diagram of sensor structure and working principle: (**a**) schematic diagram of sensor structure; (**b**) schematic diagram of working principle of triboelectric generator module; (**c**) schematic diagram of working principle of electromagnetic power generator module.

Figure 1 illustrates the structure and working principle of the sensor. As shown in Figure 1a, the sensor is installed on a potential landslide body. Its basic structure consists of two module units: a triboelectric generator and an electromagnetic generator. Two module units work independently. The triboelectric generator, characterized by its high voltage and low current, is used as the sensing unit, while the electromagnetic generator, with its larger power output, serves as the power generation module. The electromagnetic generator module mainly comprises an annular magnet and six coils. Rainwater impacts the impeller of the electromagnetic generator, causing it to rotate, which in turn rotates the annular magnet. This rotation results in the cutting of magnetic field lines by the coils, generating an induced current, thus achieving power generation. The triboelectric generator module primarily consists of a tipping bucket and a base. The tipping bucket features a triangular chamber design with a capacity of 4 mL and is fixed to the base. The material of the base is PLA. This paper selects Kapton and Cu as friction materials based on the triboelectric series, also taking into account the ease of material acquisition and processing. A Kapton film with dimensions of 25 × 20 × 0.05 mm is attached to the back of the tipping bucket as a triboelectric layer. Correspondingly, a copper film with a diameter of 20 mm and a thickness of 0.1 mm is attached to the base at the friction location aligned with the tipping bucket, serving as the other triboelectric layer. These two triboelectric layers form a single-electrode triboelectric nanogenerator. An EPE foam is adhered to the back of the tipping bucket, providing excellent elastic–plastic deformation capability. This allows for deformation during contact and compression with the base, ensuring sufficient contact between the Kapton and copper triboelectric layers. When rainwater, having passed through the electromagnetic generator, further enters the tipping bucket of the triboelectric generator, the tipping bucket will rotate as the water volume increases. This rotation causes the Kapton and copper triboelectric layers to rub against each other, generating a triboelectric signal, which simultaneously achieves power generation and tipping bucket count measurement. Since the volume of the tipping bucket is fixed at 4 mL, each generated triboelectric signal signifies that the bucket has collected 4 mL of water. Therefore, the time interval between two consecutive triboelectric signals can be recorded by the subsequent circuit, and by combining this interval with the bucket volume, the average rainfall rate during this period can be calculated. Furthermore, the sensor does not include subsequent circuitry. Users can design specific signal processing and power management circuits based on their actual measurement and power generation functional requirements.

The detailed working principles of the triboelectric generator and the electromagnetic generator are further explained below concerning Figure 1b,c. As shown in Figure 1b-S1, in the initial state, the Kapton and Cu triboelectric layers are in full contact. Based on the electron-donating and accepting capabilities of the nanomaterials in the triboelectric series, the Kapton surface carries negative charges, while the Cu surface carries an equal amount of positive charges. As the device operates, progressing to the state shown in Figure 1b-S2, the movement of the tipping bucket causes the Kapton triboelectric layer to gradually separate from the Cu triboelectric layer. At this time, due to the weakening attraction between the negative charges on the Kapton surface and the positive charges on the Cu surface, electrostatic induction occurs on the Cu triboelectric layer. Negative charges are transferred from the ground to the Cu triboelectric layer, generating an induced current in the circuit. When the Kapton is completely separated from the Cu, as shown in Figure 1b-S3, the charge transfer in the Cu triboelectric layer is complete, and no induced current is generated in the circuit. As the Kapton moves in the reverse direction, reaching the state shown in Figure 1b-S4, under the attraction of the negative charges on the Kapton, the charges in the Cu triboelectric layer are transferred back to the ground, generating a reverse transfer current in the circuit. Therefore, within one working cycle, a triboelectric pulse signal is generated in the circuit. The frequency of the triboelectric charge pulse signal corresponds one-to-one with the number of triboelectric contacts, enabling the dual functions of power generation and rainfall monitoring.

In the electromagnetic generator unit, the fan blades and the ring magnet are rigidly fixed together. When the rainwater flow drives the fan blades to rotate, the ring magnet also rotates coaxially. As shown in Figure 1c, the relative motion between the coil and the ring magnet causes a change in the magnetic flux passing through the coil. According to Faraday’s law of electromagnetic induction, the change in magnetic flux induces an electromotive force (EMF) in the coil, thereby generating a current. The direction of the induced current can be determined using Lenz’s law and the right-hand screw rule. Specifically, since the direction of the induced current always attempts to oppose the change in magnetic flux, when the ring magnet rotates to the states shown in Figure 1c-S1 and 1c-S2, the direction of the induced current in the coil is clockwise and counterclockwise, respectively. This results in a continuously changing alternating current (AC) being generated in the electromagnetic generator, thus achieving the function of power generation.

## 3. Experiments and Analysis

**Figure 2:**
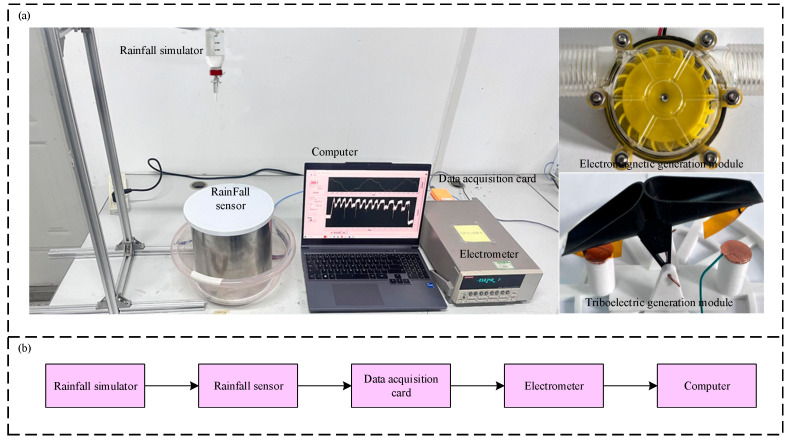
Test device: (**a**) experimental setup; (**b**) data processing flowchart section may be divided by subheadings. It should provide a concise and precise description of the experimental results, their interpretation, as well as the experimental conclusions that can be drawn.

To investigate the sensing and power generation performance of the rainfall sensor under varying rainfall intensities, an indoor simulated experimental setup was constructed, as depicted in Figure 2. This experimental setup primarily consists of a rainfall simulator, a rainfall sensor, a data acquisition card, an electrometer, and a computer. During the experiment, the rainfall simulator precisely controls the time interval between water droplet release and the water droplet size, thereby simulating different rainfall intensities. Water droplets, introduced through the water inlet, initially reach the electromagnetic generator module, inducing power generation. Subsequently, the water flows into the triboelectric generator module, inducing triboelectric generation. The electrical signals generated by the sensor are read by both the data acquisition card and the electrometer before being transmitted to the computer. A LabVIEW-basedsoftware program (version 13.0) installed on the computer enables real-time display of the measured data. The measurement function, power generation function, and environmental adaptability of the sensor were tested, as detailed below.

### 3.1. Measurement Function Tests and Analysis

**Figure 3:**
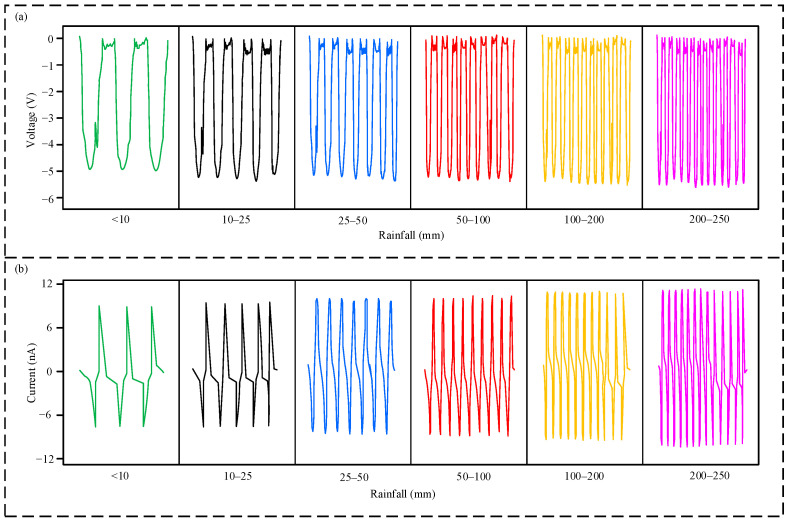
Output characteristics of the triboelectric nanogenerator (TENG) module: (**a**) output voltage waveforms at different rainfall intensities; (**b**) output current waveforms at different rainfall intensities.

Figure 3 shows the output voltage and current waveforms of the sensor’s triboelectric nanogenerator (TENG) module under different rainfall intensities. Rainfall intensity is classified according to the World Meteorological Organization’s precipitation intensity classification standards: rainfall less than 10 mm is defined as light rain, 20–25 mm as moderate rain, 25–50 mm as heavy rain, 50–100 mm as rainstorm, 100–200 mL as heavy rainstorm, and 200–250 mm as exceptionally heavy rainstorm. As shown in Figure 3a, the frequency of the output voltage waveform gradually increases with increasing rainfall intensity, while the output voltage amplitude does not exhibit a significant change, remaining approximately at −5 V. Figure 3b shows the output current variation. As rainfall intensity increases, the frequency of the output current waveform gradually increases, and the current amplitude also increases slightly, with a maximum output current of approximately 11.5 nA. This phenomenon occurs because the output voltage of the TENG is related to the contact area of the triboelectric materials. As the rainfall intensity increases, the triboelectric contact area of the sensor does not change significantly; therefore, the voltage amplitude remains relatively constant. However, the output current is related to the amount of charge transfer per unit time. As the rainfall intensity increases, the frequency of the sensor’s tipping bucket rotation increases, leading to an increase in the amount of charge transfer per unit time. Consequently, the output current increases slightly.

**Figure 4:**
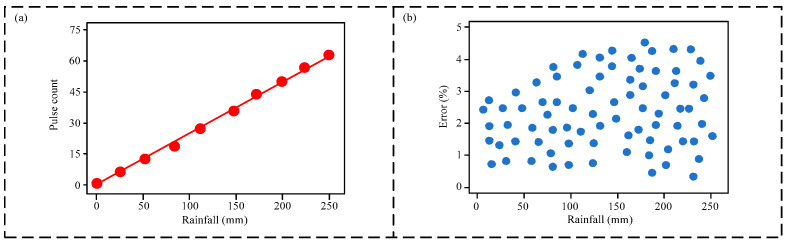
Sensor performance evaluation: (**a**) calibration curve of the triboelectric nanogenerator (teng) module; (**b**) measurement error scatter plot.

Figure 4 shows the calibration curve and measurement error scatter plot for the triboelectric nanogenerator (TENG) module. As shown in Figure 4a, the number of output pulses from the TENG module increases gradually with increasing rainfall intensity, exhibiting a good linear relationship. This indicates that the sensor possesses a satisfactory measurement function. To further assess the sensor’s measurement error at different rainfall intensities, a total of 5000 experimental data points were collected. A representative measurement error scatter plot is shown in Figure 4b. The measurement error exhibits a relatively wide range of fluctuation, but the maximum measurement error is less than 5%. Therefore, the sensor’s measurement error is defined as 5%.

### 3.2. Power Generation Performance Tests and Analysis

**Figure 5:**
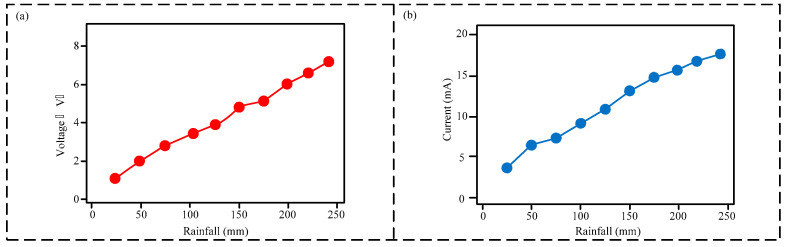
Output characteristics of the electromagnetic generator (EMG) module: (**a**) output voltage vs. rainfall intensity with linear fit; (**b**) output current vs. rainfall intensity with linear fit.

The primary function of the electromagnetic generator (EMG) module is power generation. Therefore, the output voltage and current of the EMG module were tested under varying rainfall intensities. As shown in Figure 5a, the output voltage of the EMG module increases linearly with increasing rainfall intensity, rising from 0.5 V to 6.3 V. Similarly, Figure 5b demonstrates that the output current of the EMG module also increases linearly with increasing rainfall intensity, increasing from 5 mA to 17.5 mA. This phenomenon is attributed to the increased rotational speed of the EMG module’s blades at higher rainfall intensities, resulting in a greater rate of magnetic flux cutting per unit time, thereby producing a higher output current and voltage.

**Figure 6:**
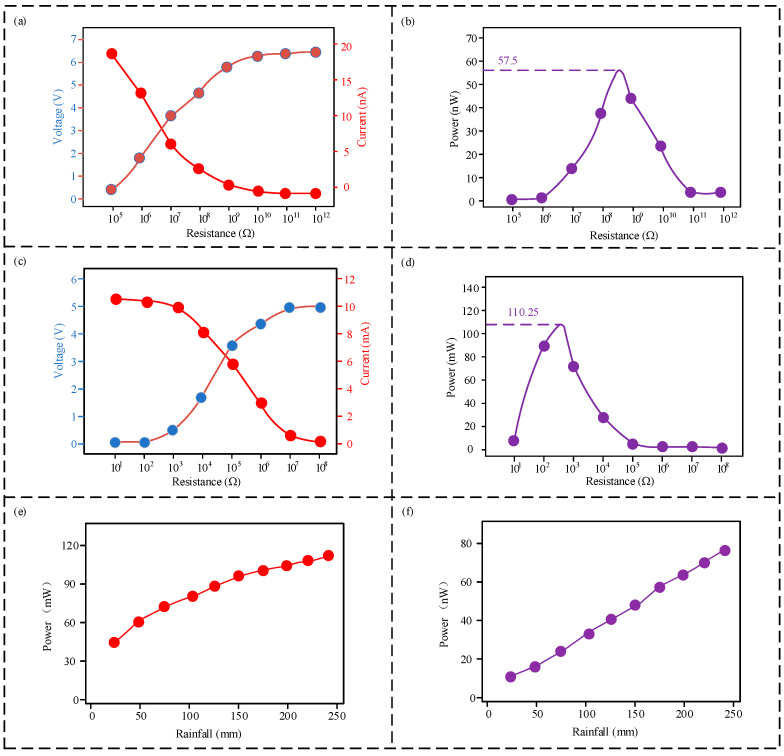
Power generation performance of sensor modules under varying external loads: (**a**) output voltage and current of the triboelectric nanogenerator (TENG) module; (**b**) output power of the TENG module; (**c**) output voltage and current of the electromagnetic generator (EMG) module; (**d**) output power of the EMG module; (**e**) linear fitting of EMG module output power and rainfall intensity; (**f**) linear fitting of TENG module output power and rainfall intensity.

Both the triboelectric nanogenerator (TENG) and electromagnetic generator (EMG) modules of the sensor possess power generation capabilities. Consequently, the output characteristics of both modules were tested under varying external loads at a constant rainfall intensity of 180 mm. The results are presented in Figure 6. As shown in Figure 6a,b, the output voltage of the TENG module increases gradually with increasing external load while the output current decreases. The maximum output power of 57.5 nW is achieved at an external load of 4.3 × 10^8^ Ω. Similarly, as shown in Figure 6c,d, the output voltage of the EMG module increases gradually with increasing external load while the output current decreases. The maximum output power of 110.25 mW is achieved at an external load of 3.6 × 10^2^ Ω. As shown in Figure 6e,f, the output power of both the EMG and TENG modules increases with increasing rainfall.

### 3.3. Environmental Adaptability Tests and Analysis

Given that the rainfall sensor is intended for long-term outdoor deployment, it is essential to investigate the influence of environmental temperature and humidity variations on the output signals of each sensor module to ensure long-term stable and reliable operation. Temperature and humidity experiments were conducted using a controlled temperature chamber and humidifier, respectively. During the experiments, the sensor was placed inside the temperature chamber. For the temperature experiments, the humidity was maintained at ambient (room) humidity, and the temperature was varied manually by adjusting the temperature chamber settings. Similarly, for the humidity experiments, the temperature was maintained at room temperature, and the humidity was varied manually by adjusting the humidifier settings.

**Figure 7:**
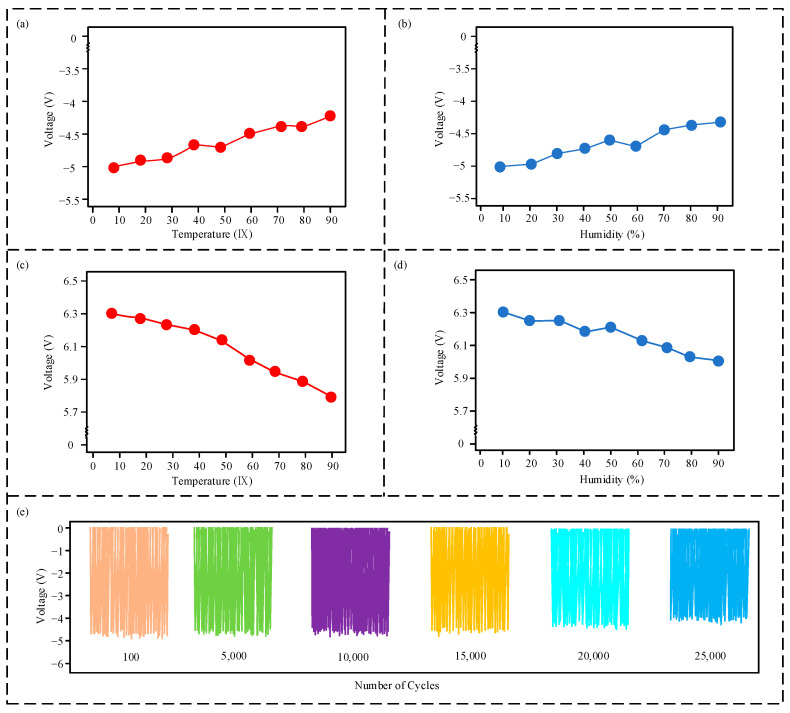
Environmental adaptability test results: (**a**) output voltage of the triboelectric nanogenerator (TENG) module vs. temperature; (**b**) output voltage of the TENG module vs. humidity; (**c**) output voltage of the electromagnetic generator (EMG) module vs. temperature; (**d**) output voltage of the EMG module vs. humidity; (**e**) output voltage of the TENG module vs. number of cycles.

As shown in Figure 7a, the output voltage of the triboelectric nanogenerator (TENG) module decreases from −5 V to −4.23 V as the temperature increases from 0 °C to 90 °C, representing a reduction of 15.5%. Similarly, Figure 7b shows that the output voltage of the TENG module decreases from −5 V to −4.32 V as the humidity increases from 0% to 90%, corresponding to an attenuation rate of 13.7%. Correspondingly, as shown in Figure 7c,d, the output voltage of the electromagnetic generator (EMG) module decreases by 7.7% and 5.1% within the temperature range of 0 °C to 90 °C and the humidity range of 0% to 90%, respectively. The resulting voltage amplitudes are 5.81 V and 5.98 V, respectively. These results indicate that the TENG module is more susceptible to the effects of temperature and humidity than the EMG module.

For the sensor, the output signal of the TENG module is used as the detection signal. Because the TENG output signal is pulsed, the subsequent microprocessor typically connects directly to the sensor output signal via a pulse input port. As the pulse input port adheres to the Transistor–Transistor Logic (TTL) logic level standard, signals with a voltage amplitude greater than 2 V are considered valid high-level signals. Therefore, even after temperature and humidity variations, the output signal voltage amplitude of the sensor remains significantly greater than the TTL high-level signal threshold, ensuring a valid signal and, thus, no impact on the sensor’s detection. Consequently, the operating temperature range of the sensor can be defined as 0 °C to 90 °C, and the operating humidity range can be defined as 0% to 90%.

Furthermore, the signal stability of the sensor during long-term operation was tested. Because the EMG module has no frictional contact components, while the frictional contact of the TENG module can degrade the signal output performance, only the TENG module was tested for long-term stability. As shown in Figure 7e, after 25,000 cycles, the output voltage decreases to approximately −4 V, which remains above the TTL voltage threshold for effective signal recognition. This demonstrates that the sensor exhibits good long-term operational stability.

### 3.4. Field Experimentation, Testing, and Analysis

**Figure 8:**
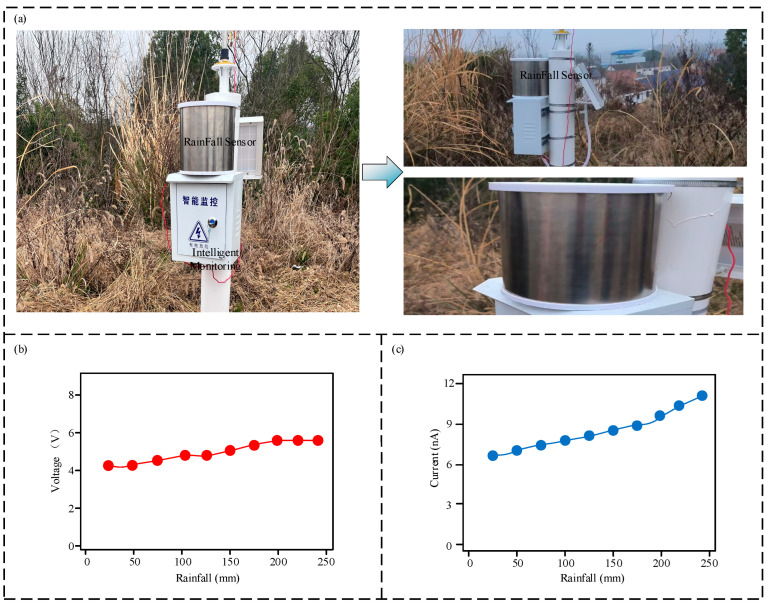
Field experimental testing: (**a**) physical objects on site. (**b**) output voltage waveforms under different on-site rainfall intensities. (**c**) output current waveforms under different on-site rainfall intensities.

Figure 8 shows the on-site test setup. As shown in Figure 8a, which is a photograph of the actual on-site setup, the rainfall sensor collects rainwater. Figure 8b,c present the on-site data. It is evident that as the rainfall amount increases, both the voltage amplitude and current amplitude of the sensor’s output signal progressively increase. The increase in voltage amplitude is relatively small, while the increase in current amplitude is considerably larger. This is consistent with the experimental results.

## 4. Conclusions and Discussions

This study presents a hybrid landslide rainfall sensor based on triboelectric nanogenerator (TENG) and electromagnetic generator (EMG) technologies, capable of simultaneously measuring rainfall and providing real-time power generation. Experimental results indicate that the sensor exhibits a measurement error of less than 5% and can operate stably in environmental conditions with temperatures below 90 °C and humidity levels below 90%. Furthermore, the sensor demonstrates power generation capabilities, with the TENG module delivering a maximum power output of 57.5 nW when connected to a 4.3 × 10^8^ Ω load resistor and the EMG module delivering a maximum power output of 110.25 mW when connected to a 3.6 × 10^2^ Ω load resistor.

Compared to conventional rainfall sensors, this sensor integrates power generation functionality. The combined TENG and EMG architecture further enhances power generation capabilities, enabling self-powered operation, which is particularly advantageous for remote landslide monitoring applications. However, the detection accuracy of the sensor requires further improvement. Future work will focus on optimizing the sensor structure or exploring the use of high-performance nanomaterials to enhance the output voltage amplitude, thereby increasing the signal-to-noise ratio and reducing the occurrence of false positive detections, which will ultimately improve the measurement precision. Furthermore, designing a suitable data processing circuit to store the electrical energy generated by TENG is also expected to power subsequent circuit boards.

## Data Availability

The original contributions presented in the study are included in the article; further inquiries can be directed at the corresponding author.
